# The role of Cdk5 in neurological disorders

**DOI:** 10.3389/fncel.2022.951202

**Published:** 2022-07-28

**Authors:** Chuncao Ao, Chenchen Li, Jinlun Chen, Jieqiong Tan, Liuwang Zeng

**Affiliations:** ^1^Department of Neurology, Second Xiangya Hospital, Central South University, Changsha, China; ^2^Center for Medical Genetics, School of Life Sciences, Central South University, Changsha, China; ^3^Hunan Key Laboratory of Medical Genetics, Central South University, Changsha, China; ^4^Hunan Key Laboratory of Animal Model for Human Diseases, Central South University, Changsha, China

**Keywords:** cyclin-dependent kinases 5 (Cdk5), neurological disorders, therapeutic targets, CDK5 inhibitor, nervous system

## Abstract

Neurological disorders are a group of disorders with motor, sensory or cognitive damage, caused by dysfunction of the central or peripheral nervous system. Cyclin-dependent kinases 5 (Cdk5) is of vital significance for the development of the nervous system, including the migration and differentiation of neurons, the formation of synapses, and axon regeneration. However, when the nervous system is subject to pathological stimulation, aberrant activation of Cdk5 will induce abnormal phosphorylation of a variety of substrates, resulting in a cascade signaling pathway, and thus lead to pathological changes. Cdk5 is intimately related to the pathological mechanism of a variety of neurological disorders, such as A-β protein formation in Alzheimer’s disease, mitochondrial fragmentation in cerebral ischemia, and apoptosis of dopaminergic neurons in Parkinson’s disease. It is worth noting that Cdk5 inhibitors have been reported to have neuroprotective effects by inhibiting related pathological processes. Therefore, in this review, we will briefly introduce the physiological and pathological mechanisms of Cdk5 in the nervous system, focusing on the recent advances of Cdk5 in neurological disorders and the prospect of targeted Cdk5 for the treatment of neurological disorders.

## Introduction

Neurological disorders are featured by impairment movement, sensation, cognition, and behavior, which is caused by damage to the peripheral nervous system or central nervous system. These diseases share similar pathophysiological mechanisms, such as oxidative stress, cytotoxicity, mitochondrial fragmentation, autophagy, endoplasmic reticulum stress, neuroinflammation, and calcium overload. However, divergent pathological manifestations exist in the different disorders (Sorensen, 2019). Cyclin-dependent kinase 5 (Cdk5) is a proline-directed serine/threonine kinase, which is a unique component of the family of cyclin-dependent kinases (Dhavan and Tsai, 2001; Malumbres, 2014). Cdk5 plays a pivotal role in the nervous system, including cortex layer formation, synaptic growth and maturation, synaptic vesicular transport (Liu et al., 2022; Takahashi et al., 2022a), stress-enhanced memory consolidation, dendritic spine formation, neuronal migration and differentiation, neurite outgrowth and length (Chen et al., 2017; Huang et al., 2017; Shinmyo et al., 2017; Lee et al., 2018; Nishino et al., 2019; Rao et al., 2020; Im et al., 2022), learning and long-term behavioral changes, axonal regeneration (Xu et al., 2017; Hwang and Namgung, 2021), brain microtubule network and actin cytoskeleton remodeling (Shah and Lahiri, 2017; Shah and Rossie, 2018), as well as normal cerebellar development and functions (Lee et al., 2019; Li et al., 2019; Kodani et al., 2020; Ouyang et al., 2020). Additionally, Cdk5 also plays a key role in gene expression, cell differentiation, angiogenesis, and aging (Arif, 2012).

Cdk5 plays an important role in the pathological process of neurological diseases. Cdk5 binds to specific partners p35 and p39, after the pathological stimulus. P35 and p39 are cleaved to p25 and p29 by calpain, with the increase in calcium concentration. The association of Cdk5/p25 is more stable and leads to aberrant hyperphosphorylation of substantial Cdk5 substrates, resulting in cell death or apoptosis (Asada et al., 2012; Nie et al., 2022). Cdk5 has also been implicated in the development and progression of a variety of cancers, including breast, lung, colon, pancreatic, melanoma, thyroid, and brain tumors, making it a promising drug target for new anticancer treatments (Pozo and Bibb, 2016).

When Cdk5 inhibitors are given, they show neuroprotective effects on numerous cell and animal models. Cdk5 inhibitors have great potential to be a therapeutic target for neurological system diseases. In this review, we will summarize recent advances in the molecular mechanisms of Cdk5 in neurological diseases as well as the therapeutic potential of Cdk5 in these neurological diseases.

**Figure 1:**
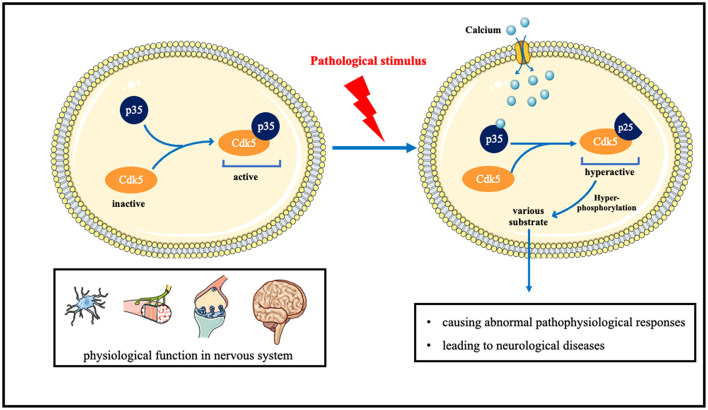
The role of Cdk5 in neurological disorders and the underlying molecular mechanisms.

Under normal circumstances, Cdk5 is in an inactive state. After binding to p35, it is normally activated, phosphorylates many substrates, and plays normal physiological functions such as neuron development and development, axonal dendrite growth, and prominent functions. When neurons are pathologically stimulated, the influx of intracellular calcium ions increases, and after combining with calcium, p35 is split into p25. The combination of p25 and CDK5 will cause CDK5 to be in an over-activated state, thereby hyperphosphorylating various substrates in cells, causing abnormal pathophysiological responses, and leading to neurological diseases (Figure 1).

## Neurodegenerative Diseases

Cdk5 hyperactivation contributes to several neurodegenerative diseases, including Alzheimer’s disease (AD), Parkinson’s disease (PD), and Huntington’s disease (HD; Cheung and Ip, 2012).

### Alzheimer’s disease (AD)

#### Aβ generation

Aβ protein deposition is not only the pathological feature of AD but also the key factor of AD progression. Aβ is produced through sequential amyloidogenic cleavage of precursor protein APP by β-secretase BACE1 and γ-secretase, with the oligomer form considered to be the toxic form (Mawuenyega et al., 2010). The mechanisms underlying Cdk5 in Aβ generation and neurotoxicity are multifaceted. Cdk5 activation promotes Aβ generation and accumulation in neurons. PPAR-β, a nuclear receptor with a key role in metabolic processes, is a phosphorylated substrate of Cdk5 and involved in Aβ generation (Quan et al., 2019; Ribeiro Filho et al., 2019). Hypoxia leads to abnormal phosphorylation of Cdk5 and accelerates the process of AD (Fang et al., 2019). Cdk5/HIF-1 contributes to Aβ generation (Chao et al., 2020). Cdk5 regulates Aβ-induced mitochondrial fission and neurotoxicity by phosphorylating Drp1 (Guo et al., 2018), which may be mediated by P27 (Jaiswal and Sharma, 2017). APP, GSK-3 β, and TrkA play important roles in Aβ pathological process, which is at least partly mediated by Cdk5 activation (Palop and Mucke, 2010). Aβ regulates the activity of Cdk5 as well (Lapresa et al., 2019). Aβ protein physiological aggregation regulates Cdk5/Calcineurin signaling (Lazarevic et al., 2017). Therefore, inhibiting the activity of Cdk5 is of great significance in reducing Aβ generation and delaying the progress of AD. Roscovitine, an inhibitor of Cdk5, effectively reverses Aβ formation. Ginsenoside Rg1 can decrease Aβ level *via* inhibiting the Cdk5/ PPAR γ pathway (Quan et al., 2020). Some other drugs, such as streptozotocin, ketamine, and phosphocreatine, also decrease Aβ levels through Cdk5 related pathway (Li et al., 2020a; Park et al., 2020; Ai et al., 2022). Therefore, targeting Cdk5 and the related pathways is a potential strategy for AD therapy.

#### Tau phosphorylation

Tau phosphorylation and the formation of neurofibrillary tangles in neurons is one of the notable hallmarks of AD pathology. Aberrant phosphorylation and deposition of tau, which is a cellular microtubule associated protein, will affect the activity of microtubule and destroy cytoskeleton structure, leading to neuronal apoptosis. Tau is a substrate of different kinases, such as Cdk5, GSK-3β, or PKA. Cdk5 plays a key role in tau phosphorylation and neurofibrillary tangles formation (Seo et al., 2017). The miR-148a-3p/p35/PTEN signaling pathway is an important pathway for tau hyperphosphorylation in AD (Zeng et al., 2021). Many factors such as MARK4 (Saito et al., 2019), and RPS23RG1 (Zhao et al., 2021), have been proved to be related to tan pathology through regulating Cdk5 activity. Glutamate induces Cdk5 and p35 mRNA transcription. The glutamate-responsive increase of the Cdk5/p25 complex contributes to tau hyperphosphorylation (Tanaka et al., 2022). Cdk5 is also associated with early inflammation of AD (Wilkaniec et al., 2018). Leukotriene, an inflammatory factor, may accelerate tau pathological accumulation through the Cdk5 pathway (Giannopoulos et al., 2019). Tau affects the function of subcellular organelles, such as mitochondria and Golgi apparatus, through Cdk5 (Mohamed et al., 2017). Cdk5 inhibitory peptide (CIP) can not only prevent the loss of neurons and alleviate behavioral changes but also reduce tau hyperphosphorylation and inflammation (Xu et al., 2019; Huang et al., 2020). Drugs like Quercetin can inhibit the pathological process of tau through the Ca^2+^-calpain-p25-Cdk5 pathway (Shen et al., 2018). For the pivotal role of Cdk5 in tau phosphorylation and the formation of neurofibrillary tangles, Cdk5 is supposed to be a promising therapeutic target for AD.

#### Cdk5 inhibition in AD

Cdk5 inhibitors can delay the pathological progression of AD and prevent neuronal apoptosis. They have not been put into clinical trials because of poor selectivity. More and more studies continue to try to find new Cdk5 inhibitors and new substrates of Cdk5 (Zhuang et al., 2020b). Recent studies have found some new substrates and signaling pathways related to Cdk5 in AD, such asCdk5-Mcl-1axis (Nikhil and Shah, 2017), ALDH1A1 (Nikhil et al., 2019), miR-125b (Zhuang et al., 2020a), and miR-504-3p (Chen et al., 2022). New Cdk5 inhibitors are also being studied in various AD models like pyrrolidine-2,3-dione, and TFP5 (Shukla et al., 2017; Zeb et al., 2019a, b). It is also found that a traditional Chinese medicine, Nano-HO, improves cognitive function in AD by modulating the signaling pathway JNK/cdk5/GSK-3β (Qu et al., 2021). Similarly, Kaixinsan, a traditional Chinese medicine for insomnia, is found to attenuate tau hyperphosphorylation and neuroinflammation by inhibiting GSK3β and CDK5 activation (Jiao et al., 2022). All of these chemicals have shown neuroprotective effect but the specific mechanism is still not fully elucidated.

### Parkinson’s disease (PD)

The main pathological change of Parkinson’s disease is the degeneration of substantia nigra pars compacta neurons. However, the mechanism of Lewy body formation is still unclear. Moreover, there is no effective treatment to slow down the process of neurodegeneration (Kalia and Lang, 2015). Dysregulation of Cdk5 is supposed to be related to the loss of dopaminergic neurons and the progression of PD. Aberrant p25/Cdk5 signaling was found in early-stage PD (He et al., 2020). Previous studies found that Cdk5 can promote oxidative stress, and lead to mitochondrial dysfunction and autophagy dysfunction in PD. Moreover, in the mouse model of PD induced by MTPP, aberrant Cdk5 results in activation of inflammation (Cheng et al., 2020), immune hyperactivity (Shukla et al., 2019), mitochondrial fission (Park et al., 2019), and degradation of ubiquitin ligases (Wang et al., 2018), leading to the loss of dopaminergic neurons. Cdk5 phosphorylation induced nuclear translocation of SIRT2 also leads to the loss of dopaminergic neurons (Yan et al., 2022). Similarly, Cdk5 inhibitors have exerted neuroprotective effects in PD (He et al., 2018). Luteolin was reported to confer neuroprotective effect on the PD model, which was also mediated by Cdk5 (Reudhabibadh et al., 2021). Therefore, based on previous studies, Cdk5 has been demonstrated to play a critical role in the development of PD, thus making it to be a pivotal target for PD therapy (Table 1).

**Table 1:** Summary of the main targets of cdk5 in neurological diseases and mechanism of Cdk5 and nervous system diseases.

		**Targets or Pathways**	**References**
**Nervous system diseases**	**Alzheimer’s disease**	**Aβ generation**: Cdk5-PPAR-β, Ginsenoside Rg1-Aβ-Cdk5/PPARγ pathway, Cdk5/Calcineurin signaling, Cdk5/Drp1-mediated mitochondrial fission, GSK-3 β and TrkA/Cdk5, Cdk5/HIF-1	**(Mawuenyega et al., 2010; Palop and Mucke, 2010; Cheung and Ip, 2012; Jaiswal and Sharma, 2017; Lazarevic et al., 2017; Guo et al., 2018; Fang et al., 2019; Lapresa et al., 2019; Quan et al., 2019, 2020; Ribeiro Filho et al., 2019; Chao et al., 2020; Li et al., 2020a; Park et al., 2020; Ai et al., 2022)**
		**Tau phosphorylation**: Leukotriene/Cdk5, Cdk5/mitochondria and Golgi function, Cdk5/MARK4, Cdk5/RPS23RG1, miR-148a-3p/p35/PTEN signaling pathway, Quercetin-Ca^2+^- calpain-p25-Cdk5 pathway, miR-504–3p and CDK5 axis	**(Mohamed et al., 2017; Shen et al., 2018; Wilkaniec et al., 2018; Giannopoulos et al., 2019; Saito et al., 2019; Xu et al., 2019; Huang et al., 2020; Zeng et al., 2021; Zhao et al., 2021; Tanaka et al., 2022)**
**Neurodegenerative diseases**		**Cdk5 inhibitor in AD**: Cdk5/Mcl-1, Cdk5-/ALDH1A1, Cdk5/miR-125b, Nano-HO-JNK/Cdk5/GSK-3β, Kaixinsan/ GSK-3β and CDK5	**(Nikhil and Shah, 2017; Shukla et al., 2017; Nikhil et al., 2019; Zeb et al., 2019a, b; Zhuang et al., 2020a, b; Qu et al., 2021; Chen et al., 2022; Jiao et al., 2022)**
	**Parkinson’s disease**	Cdk5/inflammation, Cdk5/immune hyperactivity, Cdk5/mitochondrial fission, Cdk5/degradation of ubiquitin ligases, Cdk5-Luteolin, phosphorylation induced SIRT2 nuclear translocation	**(He et al., 2018; Wang et al., 2018; Park et al., 2019; Shukla et al., 2019; Cheng et al., 2020; Reudhabibadh et al., 2021; Yan et al., 2022)**
	**Huntington’s disease**	P25/Cdk5, Cdk5-drp1, Cdk5/DARPP-32	**(Paoletti et al., 2008; Langhorne et al., 2011; Cherubini et al., 2015; Brito et al., 2019)**
	**Ischemia Stroke**	Cdk5/Zinc chelator, Cdk5/neuregulin-1 β, Cdk5/TFP5, Cdk5/tat-Cdk5 CTM, Cdk5/ERK1/2 signaling pathway, Cdk5/inhibition of p53 dependent apoptosis, Cdk5/trkb-erk1/2-creb pathway, Cdk5/phosphorylation of drp1s616, miR-148b-3p *via* CDK5R1/SIRT1	**(Becerra-Calixto and Cardona-Gomez, 2017; Cui et al., 2017; Ji et al., 2017; Liu et al., 2017; Zhao et al., 2017; Munoz-Manco et al., 2018; Tuo et al., 2018; Zhang et al., 2018; Shin et al., 2019; Zhu et al., 2019; Chen et al., 2021, 2022)**
**Cerebrovascular disease**	**Intracerebral Hemorrhage**	Cdk5-ATM signalin pathway, Cdk5/P35, Cdk5- p75NTR	**(Wu et al., 2016; Roufayel and Murshid, 2019; Zhou et al., 2019)**
**Neuropathic Pain**		Cdk5/CREB, Cdk5/PPAR γ pathway, mir-196a-5p/Cdk5 axis, Cdk5/CRMP2, Cdk5-NR2A pathway, Cdk5/TRPA1	**(Li et al., 2014, 2020; Yang et al., 2014; Chernov et al., 2018; Sulak et al., 2018; Moutal et al., 2019; Zhong et al., 2019; Gomez et al., 2020a, 2021; Zhu et al., 2021)**
	**Epilepsy**	Cdk5/mitochondrial fragmentation, Cdk5/neuroinflammation, Cdk5/endoplasmic reticulum stress, Cdk5/p38 MAPK mediated microglial response	**(Tian et al., 2008, 2010; Li et al., 2016; Kim and Kang, 2017, 2018; Liu et al., 2017; Hiragi et al., 2018; Kim et al., 2019; Fan et al., 2020; Lee and Kim, 2021)**
	**Glioblastoma**	Cdk5/PIKE-A, Cdk5/DRP1, miR-21/Cdk5, Cdk5/TP5, Cdk5/DYRK1A, Cdk5/AC1MYR2, Cdk5/TRIM59, OGT/CDK5/ACSS2 pathway	**(Liu et al., 2008; Ren et al., 2015; Xie et al., 2015; Gonzalez-Vera et al., 2016; Sang et al., 2019; Peyressatre et al., 2020a, b; Tabouret et al., 2020; Chen et al., 2021; Zhou et al., 2021; Ciraku et al., 2022)**
	**Multiple sclerosis**	Cdk5/oligodendrocytes, Cdk5/lymphocyte activation	**(Pareek et al., 2010; Luo et al., 2016, 2018)**
	**Other neurological disorders**	Cdk5/ERK1/2 pathway, Cdk5/caspase-3 pathway, Cdk5/CRMP-2, Cdk5/mitochondrial kinetic defects, Cdk5/Oxidative stress, Cdk5/endoplasmic reticulum stress, nestin-Cdk5-drp1	**(Lindqvist et al., 2017; Guo et al., 2018; Kamiki et al., 2018; Roach et al., 2018; Shi et al., 2018; Spurrier et al., 2018; Wang et al., 2018; Barrett et al., 2019; Chen et al., 2019; Liu et al., 2019; Sase et al., 2019; Li et al., 2020b; Rong et al., 2020; Shukla and Singh, 2020, 2022; Xia et al., 2020; Zhang et al., 2021; Daniels et al., 2022; Manglani and Dey, 2022; Takahashi et al., 2022b; Umfress et al., 2022; Zhou et al., 2022)**

***Abbreviations**: PPAR γ, Peroxisome proliferator-activated receptor gamma; APP, Aβ precursor protein; GSK-3 β, Glycogen Synthase Kinase-3; Drp, dynamin-related protein; MARK4, microtubule-affinityregulating kinase 4; TrkA, tropomyosin-relatedkinaseA; GSK-3 β, Glycogen synthase kinase; HIF-1, Hypoxia-inducible factor 1; RPS23RG1, The type Ib transmembrane protein; Mcl-1, myeloid-cell-leukemia-sequence-1; ALDH1A1, aldehyde dehydrogenase 1 family member A1; Fbxw7, F-box/WDrepeat-containing protein 7; TFP5, a modified truncated 24-aa peptide; DARPP-32, dopamine- and cyclic-AMP-regulated phosphoprotein of molecular weight 32,000; OGDR, The oxygen glucose deprivation reperfusion; MEF2D, myocyte enhancer factor 2D; p75 NTR, p75 neurotrophic factor receptor; ATM, Ataxia Telangiectasia Mutated; ERK1, Extracellular signal-regulated kinase 1; CREB, a transcription factor; TRPA1, transient receptor potential action channel 1; NR2A, N-methyl-D-aspartate receptor subunit 2A; NMDAR, N-methyl-D-aspartate receptor; MAPK, mitogen-activated protein kinases; TP5, a thymopentin; MTLE-HS, mesial temporal lobe epilepsy with hippocampal sclerosis; PIKE-A, Isoform A of phosphatidylinositol 3-kinase enhancer; DYRK1A, Dual-specificity tyrosine phosphorylation-regulated kinase 1A; ACSS2, acetate-dependent acetyl CoA synthetase 2; CRMP2, collapsin response mediator protein 2*.

### Huntington’s disease (HD)

**Figure 2:**
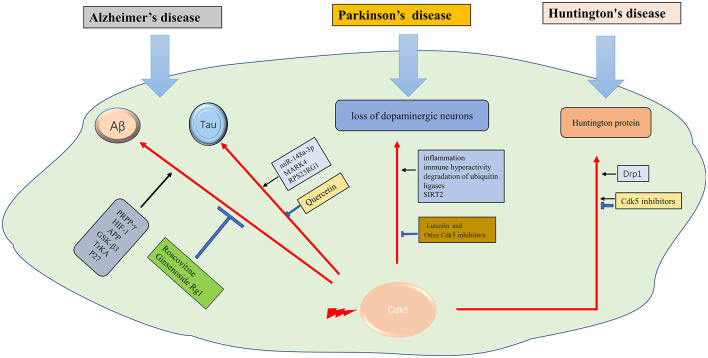
The role of Cdk5 in neurodegenerative diseases. →: promote or aggravate; ⊣: inhibit or protect.

Huntington’s disease (HD) is an autosomal dominant disease with a combination of motor, cognitive, and behavioral characteristics. HD is caused by the extended CAG trinucleotide repeat (variable length) in HTT (the gene encoding protein huntingtin; Bates et al., 2015). Huntington protein leads to neuronal dysfunction and death through a variety of mechanisms, including proteinase deposition, destruction of transcription and mitochondrial function, and direct toxicity of mutant protein (McColgan and Tabrizi, 2018). Cdk5 has been confirmed to participate in the pathological process (Bowles and Jones, 2014). It was suggested that, unlike in AD and PD, Cdk5 exerts neuroprotective effects in HD (Kaminosono et al., 2008). However, some other studies have yielded different results. It was reported that Cdk5 knockout with overexpression of mutated huntingtin (MHTT) alleviated cortical striatal learning deficits and hippocampus dependent memory decline (Alvarez-Periel et al., 2018). P25/Cdk5 signaling is an important mediator of dopamine and glutamate neurotoxicity associated with HD (Paoletti et al., 2008). Cdk5 mediates dopaminergic neurotoxicity by regulating Drp1, which induces mitochondrial fragmentation in HD pathology (Cherubini et al., 2015). In the nucleus accumbens, Cdk5 dysfunction regulates DARPP-32 phosphorylation, which contributes to depression-like behavior in HD (Brito et al., 2019). Therefore, Cdk5 is supposed to get double-sided nature in HD diseases. How to use its beneficial side and how to prevent its detrimental side is a task worthy of consideration in the future (Figure 2).

## Cerebrovascular Disease (CVD)

Cerebrovascular disease is the most common disease in the nervous system and one of the major threats to human health and life worldwide (Langhorne et al., 2011). In recent years, there is evidence that targeting Cdk5 can protect synaptic plasticity and provide long-term neuroprotection after stroke (Gutierrez-Vargas et al., 2017).

### Ischemic stroke

Cerebral ischemia is one of the most serious public health problems worldwide (Tolonen et al., 2005). The important pathophysiological mechanisms of ischemic stroke are neuroinflammation, oxidative stress, calcium overload, mitochondrial fragmentation, and Golgi stress caused by ischemia and hypoxia (Sun et al., 2019). As a unique Cdk in the nervous system, Cdk5 has been demonstrated to play an important role in the pathological process of ischemic stroke. In a large number of animal models of cerebral ischemia or neuronal cell ischemia models, Zincchelator (Tuo et al., 2018), neuregulin-1 β (Zhang et al., 2018), tfp5 (Ji et al., 2017), and tat-Cdk5 CTM (Zhu et al., 2019) are found to have a neuroprotective effect through Cdk5 related pathways. Cdk5 inhibition with scCdk5mir astrocytes (Becerra-Calixto and Cardona-Gomez, 2017), Cdk5 RNAi-based therapy (Munoz-Manco et al., 2018) or transplanted with PTPN21 (Cui et al., 2017) also confers neuroprotection in ischemic stroke. The mechanisms underlying Cdk5 in ischemia stroke are multifaceted, such as protecting cells through the ERK1/2 signaling pathway (Zhao et al., 2017), inhibition of p53 dependent apoptosis (Shin et al., 2019), trkb-erk1/2-creb pathway (Liu et al., 2017), phosphorylation of drp1s616 (Chen et al., 2021) and sponging miR-148b-3p (Chen et al., 2022). Given that reducing the level of Cdk5 in astrocytes could protect against brain damage in cerebrovascular diseases (Becerra-Calixto et al., 2018), it is believed that Cdk5 has great potential in the treatment of ischemic stroke.

### Intracerebral hemorrhage (ICH)

Intracerebral hemorrhage (ICH) is a common cerebrovascular disorder, accompanied by a particularly high mortality. The prognosis of ICH is not satisfying. Thus, it is essential to understand the potential molecular mechanisms of ICH-induced brain injury (Wilson et al., 2015). The most serious damage of cerebral hemorrhage to neurons is due to hematoma compression and hemorrhagic inflammation related pathological changes, while most of the subcellular pathological changes are similar to ischemic stroke. Inhibition of Cdk5 activity, such as knockout of Cdk5 kinase activity (Ke et al., 2015) or glycosylated Cdk5 (Ning et al., 2017), also showed neuroprotective effect in intracerebral hemorrhage. The Cdk5-ATM signaling pathway has been demonstrated to protect neurons in the process of cerebral hemorrhage (Wu et al., 2016). Phosphorylation of p35 also attenuated neuronal apoptosis through Cdk5 (Roufayel and Murshid, 2019). Moreover, p75NTR promotedp53 dephosphorylation and induced neuronal apoptosis after intracerebral hemorrhage (Zhou et al., 2019). Therefore, Cdk5 plays an important role in intracerebral hemorrhage and is a potential therapeutic target.

## Neuropathic Pain (NP)

Neuropathic pain is caused by a lesion or disease of the somatosensory system, including peripheral fibers and central neurons (Bouhassira, 2019). The development of NP is caused by many pathophysiological mechanisms that affect pain pathways (Colloca et al., 2017). As a unique Cdk of the nervous system, Cdk5 has been proved to play an important role in the pathogenesis of NP. The role of Cdk5 in NP and its potential substrates, such as channels, proteins involved in neurotransmitter release, and receptors, were discussed in many studies (Gomez et al., 2020b). Researchers have visualized the regulation of trigeminal sensory neurons by Cdk5, showed the expression change of Cdk5 and the accumulation of calcium ions, and provided a strong basis for revealing the pathological mechanism of neuralgia (Hu et al., 2022). The level of Cdk5 and phosphorylated CRMP2 was increased in NP models, and inhibition of CRMP2 could alleviate NP (Moutal et al., 2019). Cdk5 inhibitors can inhibit neuralgia through the Cdk5-NR2A pathway (Yang et al., 2014) or attenuate the response of TRPA1 (Sulak et al., 2018). Cdk5 also plays a critical role in regulating myelin basic protein (MBP) fragment (Chernov et al., 2018), inflammatory pain (Zhu et al., 2021), and calcium channel (Gomez et al., 2020a, 2021) in NP. Cdk5 mediated cyclic AMP response element binding protein (CREB; Li et al., 2014) and regulated NP through Cdk5/PPAR γ pathway (Zhong et al., 2019). Silencing noncoding RNA H19 can relieve by inhibiting Cdk5 mediated phosphorylation of CREB (Li et al., 2020). Based on these findings, Cdk5 is supposed to be a potential target to attenuate neuralgia.

## Epilepsy

Epilepsy is a brain disease, with more than 70 million people suffering from epilepsy worldwide (Thijs et al., 2019). The pathophysiological mechanism of epilepsy is not fully clarified. There is a high incidence of comorbidity and premature mortality in patients with epilepsy (Yuen et al., 2018). It is important to understand the molecular mechanism of epilepsy in order to find new prognostic/diagnostic biomarkers. Cdk5 naturally plays an important role in the pathological process of epilepsy. Cdk5 maintains the steady-state synaptic plasticity by regulating the synaptic cascade in neurons. In the animal refractory epilepsy model, the expression of the Cdk5 gene at the transcriptional level has been proved to be abnormal (Dixit et al., 2017). Cdk5 plays different roles in different brain regions in patients with mesial temporal lobe epilepsy with hippocampal sclerosis (Banerjee et al., 2021). The occurrence and development of epilepsy are related to the blood-brain barrier. It is verified that endothelial specific Cdk5 knockout induced spontaneous seizures in mice (Liu et al., 2020). In status epilepticus, Cdk5 promotes neuronal apoptosis through excessive mitochondrial fragmentation (Kim and Kang, 2017), regulates neuroinflammation (Hiragi et al., 2018), and endoplasmic reticulum stress (Lee and Kim, 2021). After status epilepticus, Cdk5 was less expressed in CA1 cells in animal models (Kim and Kang, 2018). Roscovitine, a Cdk5 inhibitor, inhibits status epilepticus-induced neuroinflammation by regulating p38 MAPK-mediated microglial response (Kim et al., 2019). P35 and P39, Cdk5 activators, have also been shown to play a significant role in synaptic function and epileptic response (Li et al., 2016). The change of Cdk5/p35 expression in the hippocampus may play a role in epilepsy by affecting mossy fiber germination (Tian et al., 2008, 2010). It is well known that NMDAR is intimately related to epilepsy. NMDAR induced axon injury in temporal lobe epilepsy through regulating GSK-3 β and Cdk5 (Liu et al., 2017; Fan et al., 2020). However, more studies are still needed to unveil the role of Cdk5 in the pathophysiological process of epilepsy.

## Glioblastoma

**Figure 3:**
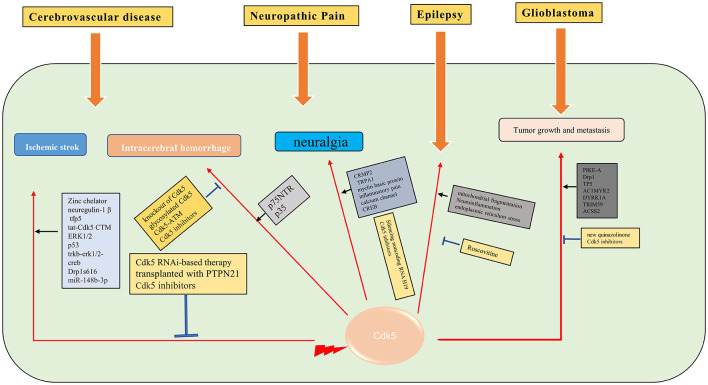
Molecular mechanisms of Cdk5 in some of the neurological diseases. →: promote or aggravate; ⊣: inhibit or protect.

Glioblastoma (GBM) is an aggressive malignant primary brain tumor. Currently, there are only palliative treatments such as radiotherapy and chemotherapy (Stupp et al., 2009). It is important to find effective therapeutic drugs for GBM. The aberrant activity of Cdk5 is found in various tumors, including GBM. A biosensor for Cdk5 can be used to probe Cdk5 activity in living glioblastoma cells by fluorescence imaging (Peyressatre et al., 2020b). The level of Cdk5 may be a potential biomarker for early diagnosis of GBM (Gonzalez-Vera et al., 2016). Cdk5 is also identified as a valuable predictive marker for tumorigenesis and progression in GBM. Cdk5 can promote the migration, invasion, and progression of GBM by phosphorylating PIKE-A (Liu et al., 2008) and Drp1 (Xie et al., 2015). Cdk5 inhibition by TP5 (Tabouret et al., 2020), AC1MYR2 (Ren et al., 2015), inhibition of DYRK1A (Chen et al., 2021), Cdk5 knockdown (Zhou et al., 2021), and a new quinazolinone family (Peyressatre et al., 2020a) can suppress the progression of GBM. Moreover, it is supposed that targeting the Cdk5/TRIM59 signal axis (Sang et al., 2019) and OGT/CDK5/ACSS2 pathway (Ciraku et al., 2022) may be future strategies for the treatment of GBM (Figure 3).

## Multiple Sclerosis

Cdk5 is essential in regulating the transformation of the precursor cells of adult oligodendrocytes to mature oligodendrocytes (Luo et al., 2014). Cdk5 plays an important role in the formation of myelin sheath for oligodendrocytes. Abnormal Cdk5 activity can lead to demyelination-related diseases, such as multiple sclerosis (Luo et al., 2016). Cdk5 activity in oligodendrocytes contributes to demyelination and cognitive dysfunction in a mouse model of multiple sclerosis (Luo et al., 2018). However, the specific mechanism for Cdk5 in multiple sclerosis is still unclear. It is found that Cdk5 can participate in the pathological process of multiple sclerosis by regulating lymphocyte activation (Pareek et al., 2010).

## Other Neurological Disorders

**Figure 4:**
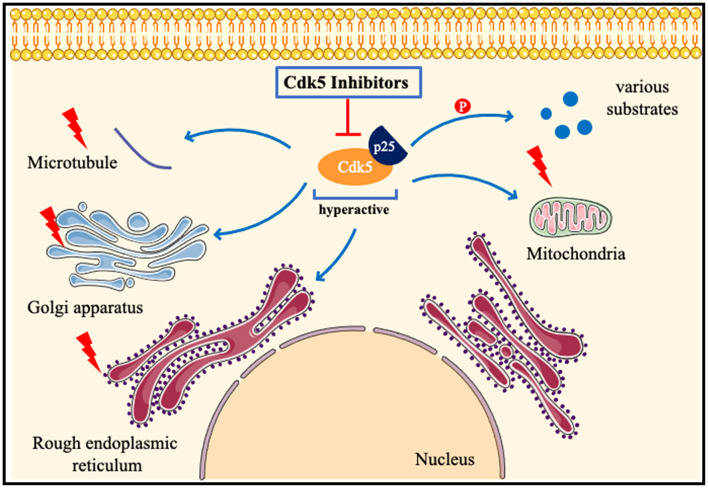
Pathologicalmechanisms of Cdk5 in neurological disorders. When neurons are subjected to pathological stimuli, such as ischemia and toxic injury. Cdk5 is highly activated after binding to p25, phosphorylates many substrates, and also induces mitochondrial fragmentation, Golgi apparatus and endoplasmic reticulum dysfunction, and destruction of the cytoskeleton. Cdk5 inhibitors can attenuate or even reverse the above pathological processes.

Cdk5 has been proved to play a key role in many other neurological diseases, such as ALS (Bk et al., 2019), early brain injury (Ding et al., 2022), cerebral amyloidosis (Kiss et al., 2020), fragile X-associated tremor/ataxia syndrome (FXTAS; Robin et al., 2017), spinal muscular atrophy (Tejero et al., 2020), and systemic sclerosis (Wei et al., 2017). Circadian behavior (Zhou et al., 2022), learning disabilities (Kamiki et al., 2018), and aging (Spurrier et al., 2018) are also closely related to Cdk5. Diabetes induces brain damage by regulating Cdk5 phosphorylation (Li et al., 2020b). Inhibition of Cdk5 improves glucose uptake in insulin-resistant neuronal cells *via* the ERK1/2 pathway (Manglani and Dey, 2022), and alleviates cognitive deficits caused by diabetes (Liu et al., 2019). Inhibition of the Cdk5/caspase-3 pathway by p5-TAT can also attenuate radiation-induced cognitive dysfunction (Zhang et al., 2021). Inhibition of Cdk5 activity ameliorates anxiety and depression in mice (Takahashi et al., 2022b). Cdk5 is found to induce mitochondrial kinetic defects in optic neuropathy (Rong et al., 2020). Cdk5 phosphorylates CRMP-2, which will aggravate optic nerve damage (Chen et al., 2019). Cdk5 contributes to oxidative stress (Guo et al., 2018) and endoplasmic reticulum stress (Shi et al., 2018). Nestin is closely related to Cdk5 signaling (Lindqvist et al., 2017) and the nestin-Cdk5-drp1 axis regulates neural stem cell stemness (Wang et al., 2018). The role of Cdk5 in nervous system diseases may be different in male and female models (Barrett et al., 2019). Estrogen promotes axon regeneration after subcortical axon injury through the PI3K/Akt/CDK5/Tau pathway (Xia et al., 2020). Epigenetic editing of Cdk5 also has been applied to neurological disorders, such as sex-specific regulation of fear memory (Sase et al., 2019). Till now, researchers try to find new phosphorylation substrates of Cdk5 (Roach et al., 2018), and discover new Cdk5 inhibitors by various biotechnology methods (Shukla and Singh, 2020, 2022). In a recent study, a highly selective inhibitor of CDK5, GFB-12811, was discovered and optimized (Daniels et al., 2022). A brain-penetrating Cdk5 inhibitor was also developed and found to alter neurobehavior (Figure 4; Umfress et al., 2022).

## Conclusion

In general, our understanding of Cdk5 in neurological disorders has made great progress in recent years. A large number of studies have confirmed its important physiological function and its toxic effect after over-activation. It is increasingly clear and certain that Cdk5 plays a pivotal role in the physiological function of the nervous system and the pathological process of neurological disorders. Cdk5 inhibitors have shown promising effects in numerous studies and Cdk5 has great potential as a therapeutic target for neurological disorders. However, many issues, such as the more detailed molecular mechanisms of Cdk5 in different neurological disorders and the development of more selective inhibitors of CDK5, still need to be further clarified before its clinical application.

## Author Contributions

CA and LZ conceived, organized, and discussed the work. CA contributed to manuscript writing and literature search. CL, JC, JT, and LZ revised the manuscript. All authors contributed to the article and approved the submitted version.

## Funding

This work has been supported by National Natural Science Foundation of China (Grant no. 81771423 and 81974212) and Natural Science Foundation of Hunan province (Grant no. 2020JJ4822).

## Conflict of Interest

The authors declare that the research was conducted in the absence of any commercial or financial relationships that could be construed as a potential conflict of interest.

## Publisher’s Note

All claims expressed in this article are solely those of the authors and do not necessarily represent those of their affiliated organizations, or those of the publisher, the editors and the reviewers. Any product that may be evaluated in this article, or claim that may be made by its manufacturer, is not guaranteed or endorsed by the publisher.
